# Gut Microbiota Dysbiosis Is Not Independently Associated With Neurocognitive Impairment in People Living With HIV

**DOI:** 10.3389/fmicb.2018.03352

**Published:** 2019-01-28

**Authors:** Fengdi Zhang, Junyang Yang, Yongjia Ji, Meiyan Sun, Jiayin Shen, Jianjun Sun, Jiangrong Wang, Li Liu, Yinzhong Shen, Renfang Zhang, Jun Chen, Hongzhou Lu

**Affiliations:** ^1^Department of Infectious Disease, Shanghai Public Health Clinical Center, Fudan University, Shanghai, China; ^2^Department of Infectious Disease, Huashan Hospital, Fudan University, Shanghai, China; ^3^Department of Internal Medicine, Shanghai Medical College, Fudan University, Shanghai, China

**Keywords:** HIV, cognitive, HIV-associated neurocognitive disorder, gut microbiota, predictive function

## Abstract

Gut microbiota dysbiosis, which has been linked to many neurological diseases, is common in HIV infection. However, its role in the pathogenesis of neurocognitive impairment is still not established. In this study, a total of 85 HIV infected subjects, naïve to antiretroviral therapy, were classified into two groups—those with HIV-associated neurological diseases (HAND) and those without, using the Montreal Cognitive Assessment (MoCA) test. Fecal samples were collected from all subjects and microbiota were analyzed by 16S rRNA amplicon sequencing. Subjects with HAND were older (*P* < 0.001), with lower levels of education (*P* = 0.002), lower CD4 T-cell counts (*P* = 0.032), and greater heterosexual preference (*P* < 0.001), than those without HAND. Gut microbiota from subjects with HAND showed significantly lower α-diversity compared to gut microbiota from subjects without HAND (Shannon index, *P* = 0.003). To exclude confounding bias, 25 subjects from each group, with comparable age, gender, CD4 T-cell count, educational level and sexual preference were further analyzed. The two groups showed comparable α-diversity (for SOB index, Shannon index, Simpson index, ACE index, and Chao index, all with *P*-value > 0.05) and β-diversity (ANOSIM statistic = 0.010, *P* = 0.231). There were no significant differences in microbiota composition between the two groups after the correction for a false discovery rate. Consistently, microbiota from the two groups presented similar predictive functional profiles. Gut microbiota dysbiosis is not independently associated with neurocognitive impairment in people living with HIV.

## Introduction

The prognosis for HIV-infected patients has improved significantly in the past two decades, as the incidence of various opportunistic infections decreased substantially due to combined antiretroviral therapy (cART) (Antiretroviral Therapy Cohort Collaboration, 2017). However, the neurological symptoms caused by HIV infection are still not well-controlled (Fauci and Marston, 2015). In 2007, these neurological symptoms were designated as HIV-associated neurocognitive disorder (HAND) (Antinori et al., 2007). A recent study reported that almost half of people living with HIV/AIDS (PLWHA) naïve to cART, demonstrated neurocognitive impairment (Robertson et al., 2018). It has been estimated that approximately 15–55% of PLWHA suffer from HAND despite effective cART (McArthur et al., 1993; Heaton et al., 2010). HIV-associated dementia (HAD), the most severe form of HAND, has been reduced from 20 to 5% as a result of broader cART coverage, while other milder forms of HAND such as asymptomatic neurocognitive disorder (ANI) and mild neurocognitive disorder (MND) remain common (Gates et al., 2016; Saylor et al., 2016). PLWHA with HAND are less able to deal with complicated activities, resulting in poor medication compliance which leads to a shorter life expectancy and a lower quality of life (Berger and Brew, 2005; Antinori et al., 2007; Tozzi et al., 2007). However, the pathogenesis of HAND remains unknown.

Growing evidence indicates that many neurological diseases, including Parkinson’s disease, Alzheimer’s disease, and multiple sclerosis, are linked to gut microbiota dysbiosis (Hindson, 2017; Wu et al., 2017; Qian et al., 2018). Abnormalities in gut microbiota may affect brain function through several mechanisms, including the modulation of the signaling pathways of the microbiota-gut-brain axis and the regulation of both the production and absorption of neurotransmitters and neurotoxic products (e.g., kynurenine downstream products) (Vecsei et al., 2013).

In HIV-infection, leaky gut and an increased plasma level of microbiota products contribute to immune activation, which has been linked to HAND (Eden et al., 2007; Sandler and Douek, 2012; Yilmaz et al., 2013; Eden et al., 2016). Furthermore, gut microbiota dysbiosis is common in HIV-infection, especially in those with low CD4 T-cell counts (Tincati et al., 2016; Ribeiro et al., 2017; Hamad et al., 2018; Williams et al., 2018). Whether gut microbiota play a role in the pathogenesis of HAND, currently remains unclear. Herein, we studied the gut microbiome of PLWHA with and without HAND to determine whether HAND is associated with differences in gut microbiota.

## Materials and Methods

### Study Settings and Design

We enrolled PLWHA at the Shanghai Public Health Clinical Center (SHPCC), Shanghai, China from September 2015 to July 2016. All PLWHA older than 18 years and naïve to cART were eligible to participate. The exclusion criteria included antibiotic/probiotic administration within 4 weeks prior to enrollment; complications including cardiopulmonary diseases, hematological diseases, malignant tumor, opportunistic infections, chronic hepatitis virus B and C infection, and syphilis; history of inflammatory bowel disease; history of central nervous system diseases or mental illness prior to HIV diagnosis; as well as pregnant women. This study was approved by the Ethics Committee of SPHCC. All subjects provided written informed consent in accordance with the Declaration of Helsinki.

Enrolled PLWHA were allocated using the Montreal Cognitive Assessment (MoCA) test (Pendlebury et al., 2012). PLWHA with scores less than 26/30 points were assigned to the HAND group, while subjects that scored greater than 26/30 points were placed in the non-HAND group.

### Sample Collection and DNA Extraction

At the time of recruitment, fecal and blood samples were collected from all subjects. The fecal samples were collected in disposable plastic sterile dung cups and properly handled. After collection, they were stored at -80°C until DNA extraction was performed using the QIAamp DNA Stool Mini Kit (Qiagen, Düsseldorf, Germany).

HIV RNA and CD4 T-cell counts were performed routinely at the clinical laboratory of the SPHCC, using Roche COBAS^®^ AmpliPrep/COBAS^®^ TaqMan^®^ HIV-1 test, version 2.0 (CAP/CTM v2.0; Roche, Basel, Switzerland) and Cytomics^TM^ FC 500 (Beckman Coulter, Brea, CA, United States) flow cytometry, respectively.

### 16S rRNA Gene Sequencing and Analysis

The targeted V3-V4 region of the bacterial 16S rRNA gene from extracted DNA, was PCR-amplified with the universal primers 341F (5′-AGA GTT TGA TCM TGG CTC AG-3′) and 805R (5′-GAC TGG AGT TCC TTG GCA CCC GAG AAT TCC A-3′) (Sakamoto et al., 2000; Chakravorty et al., 2007; Nossa et al., 2010; Haas et al., 2011). The 16S rRNA gene sequencing was performed on an Illumina MiSeq instrument (Illumina, San Diego, United States) at Sangon Biotech Co., Ltd. (Shanghai, China). All reads were demultiplexed, preprocessed, and subsequently analyzed with the Quantitative Insights into Microbial Ecology (QIIME) software package (Caporaso et al., 2010). Operational taxonomic unit (OTU) clustering was performed at a 97% similarity threshold using the QIIME pipeline. The relative abundance of the taxa at the phylum and genus levels were calculated. Alpha diversity analysis was implemented to measure the diversity of species in each sample and to calculate species diversity indices such as the SOB (the observed richness), ACE (abundance based coverage estimated), Chao, Shannon, and Simpson (Sun et al., 2016). Beta diversity was measured by unweighted and weighted UniFrac metrics and the distances were visualized by principal coordinates analysis (PCoA) (Ji et al., 2018). The OTU abundance was standardized by PICRUSt (phylogenetic investigation of communities by reconstruction of unobserved states) to conduct the 16S predictive functional analysis, that is, to remove the influence of the number of copies of the 16S marker gene in the genome of the species (Tang et al., 2018). The PICRUSt software stores the Kyoto encyclopedia of genes and genomes (KEGG) ortholog information corresponding to the greengene ID. The greengene ID obtains KEGG ortholog (KO) information corresponding to the OTU.

### Statistical Analysis

Data analysis was conducted using IBM SPSS version 20.0 software (IBM SPSS, Inc., Armonk, NY, United States) and GraphPad Prism version 5.0 (GraphPad Software, La Jolla, CA, United States). Continuous variables were described using mean and standard deviation (SD), while categorical variables were described by numbers and percentages. For characteristics analyses and diversity indices between groups, differences were estimated by the Student’s *t*-test, the Mann–Whitney *U*-test, or the chi-square test, where appropriate. All tests were two-sided and *P* < 0.05 was considered statistically significant. *P*-values were corrected to control the false discovery rate (FDR) < 0.05 using the Benjamini–Hochberg method.

## Results

### Demographic Characteristics of the PLWHA

**Figure 1:**
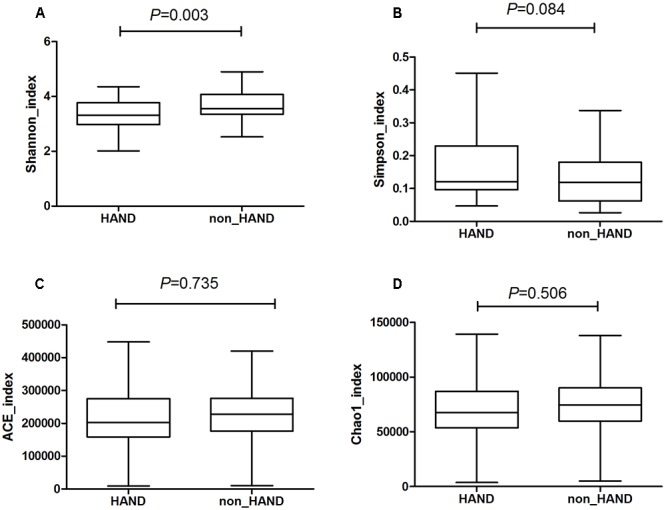
Significantly lower α-diversity of gut microbiota from HAND group compared with that from non-HAND group. **(A)** Shannon index: *t*-test; **(B)** Simpson index: Mann–Whitney test; **(C)** ACE index: Mann–Whitney test; **(D)** Chao index: *t*-test.

A total of 85 subjects were enrolled in this study. Among these 85 PLWHA, 39 were assigned to the HAND group and 46 were classified as non-HAND. PLWHA in the HAND group were older, with lower education levels, lower CD4 T-cell counts, and greater heterosexual preference than those in the non-HAND group (Table 1). As PLWHA in the HAND group had lower CD4 T-cell counts and greater heterosexual preference compared with those in the non-HAND group, the gut microbiota in the HAND group also showed significantly lower α-diversity when compared with that in the non-HAND group, as expected (Figure 1).

**Table 1:** Demographics and clinical characteristics of the total participants.

	Over all (*n* = 85)	HAND (*n* = 39)	Non-HAND (*n* = 46)	*P*-value^a^
Age (IQR, Year)	33 (27–41)	38 (31–53)	30 (26–34)	<0.001^b^
Gender (male, %)	80 (94.1%)	35 (89.7%)	45 (97.8%)	0.115^c^
Years of education (IQR)	15 (12–16)	12 (12–16)	16 (15–16)	0.002^c^
Marital status (Married or cohabiting, %)	31 (36.5%)	22 (56.4%)	9 (19.6%)	<0.001^c^
Sexual preference (homosexual, %)	68 (80%)	25 (64.1%)	43 (93.5%)	<0.001^c^
CD4 T cell counts (IQR, cells/mm^3^)	295 (199–404)	258 (187–370)	328 (228–423)	0.033^d^
HIV-RNA (IQR, Log10 copies/ mL)	4.6 (3.8–5.0)	4.5 (3.6–5.1)	4.6 (4.0–4.9)	0.148^c^
MoCA score (IQR)	26 (24–28)	24 (21–25)	28 (27–28)	<0.001^b^

*IQR, Interquartile range; NA, Not applicable. ^*a*^Compared between HAND group and non-HAND group. ^*b*^Analyzed by Mann–Whitney *U*-test. ^*c*^Analyzed by chi-square test. ^*d*^Analyzed by *t*-test*.

**Figure 2:**
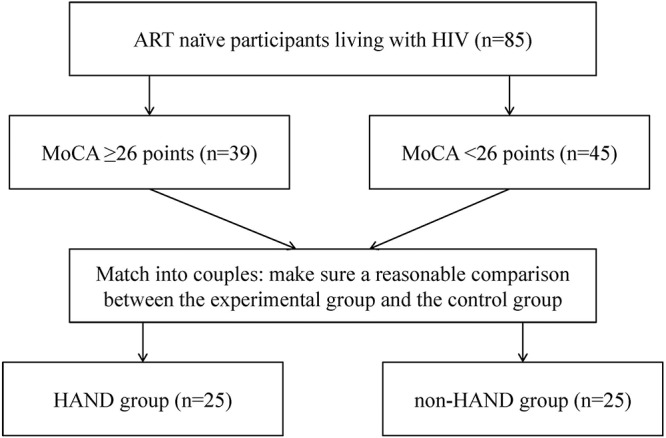
Flowchart of recruitment and grouping method.

To limit confounding bias, we matched the two groups for age, gender, immunological stage, education level, and sexual preference in 50 subjects that underwent further analysis, with 25 subjects in each group. A flowchart describing the recruitment and grouping methods of all PLWHA is shown in Figure 2.

The demographic characteristics of these subgroups are shown in Table 2. All 50 PLWHA were male, aged 33.5 (28.5–38), with 15 (12–16) years of education. The majority (86%) of the group had a homosexual preference and modest immunosuppression [CD4 T-cell counts 254 (196–353) cells/mm^3^] (Table 2). The MoCA scores in the HAND and non-HAND groups were 24 (23–25) and 28 (26.5–28.5), respectively.

**Table 2:** Demographics and clinical characteristics of participants in the subgroup analysis.

	Over all (*n* = 50)	HAND (*n* = 25)	Non-HAND (*n* = 25)	*P*-value^a^
Age (IQR, Y)	34 (29–38)	34 (30–42)	31 (27–37)	0.236^b^
Gender (male, %)	50 (100%)	25 (100%)	25 (100%)	NA
Years of education (IQR)	15 (12–16)	15 (12–16)	15 (15–16)	0.333^c^
Marital status (married or cohabiting, %)	20 (40%)	12 (48%)	8 (32%)	0.248^c^
Sexual preference (homosexual, %)	43 (86%)	20 (80%)	23 (92%)	0.221^c^
CD4 T cell counts (IQR, cells/mm^3^)	254 (196–353)	258 (211–363)	249 (189–358)	0.782^d^
HIV-RNA (IQR, Log10 copies/ mL)	4.6 (3.8–5.0)	4.4 (3.6–4.9)	4.7 (4.5–5.1)	0.747^c^
MoCA score (IQR)	25.5 (24–28)	24 (23–25)	28 (26.5–28.5)	<0.001^b^

*IQR, Interquartile range; NA, Not applicable. ^*a*^Compared between HAND group and non-HAND group. ^*b*^Analyzed by Mann–Whitney *U*-test. ^*c*^Analyzed by chi-square test. ^*d*^Analyzed by *t*-test*.

### Gut Microbiota Diversity Index

**Figure 3:**
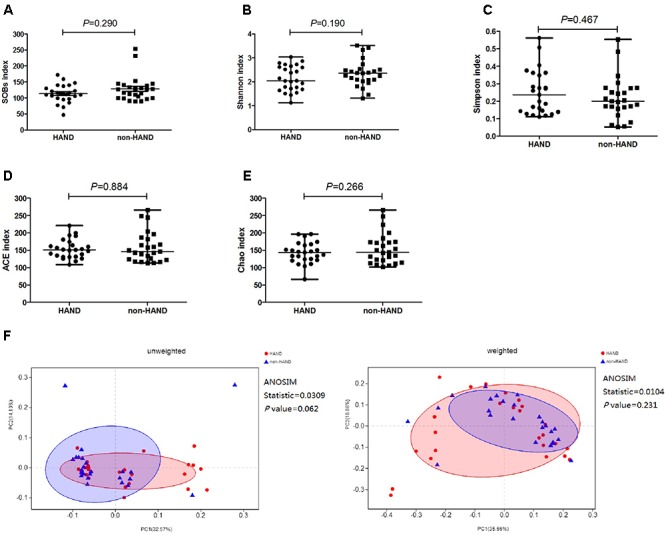
Differences in species α-diversity of gut microbiota between HAND and non-HAND groups in subgroup analysis. **(A)** SOBs index, Mann–Whitney test; **(B)** Shannon index, *t*-test; **(C)** Simpson index, Mann–Whitney test; **(D)** ACE index, Mann-Whitney test; **(E)** Chao index, *t*-test. **(F)** Unweighted and weighted analyses of similarities(ANOSIMs) and principal coordinates analysis(PCOA) based on the distance matrix of UniFrac dissimilarity of the fecal microbial communities in HAND group and non-HAND groups. Each symbol represented a sample, HAND group (red circle), non-HAND group (blue triangle). ANOSIM statistic showed the community variation between the compared groups, and *P*-values were indicated.

We compared the α-diversity by SOB, Shannon, Simpson, ACE, and Chao indices, respectively, between the two groups. The species α-diversity of gut microbiota was not significantly different between the HAND and non-HAND groups [SOB index (median 112.0 vs. 127.0, *P* = 0.290), Shannon index (median 2.044 vs. 2.356, *P* = 0.190), Simpson index (median 0.236 vs. 0.200, *P* = 0.467), ACE index (median 151.2 vs. 146.4, *P* = 0.884), and Chao index (median 143.6 vs. 144.0, *P* = 0.266) Figure 3A–E].

Furthermore, the species β-diversity of fecal microbiota was also not significantly different between the HAND and non-HAND groups (Figure 3F). We evaluated β-diversity based on the unweighted (qualitative, ANOSIM statistic = 0.031, *P* = 0.062) and the weighted (quantitative, ANOSIM statistic = 0.010, *P* = 0.231) UniFrac distance matrix of the differences between groups in the fecal microbial communities.

### No Differences in Gut Microbiota Composition Between the HAND and Non-HAND Groups

**Figure 4:**
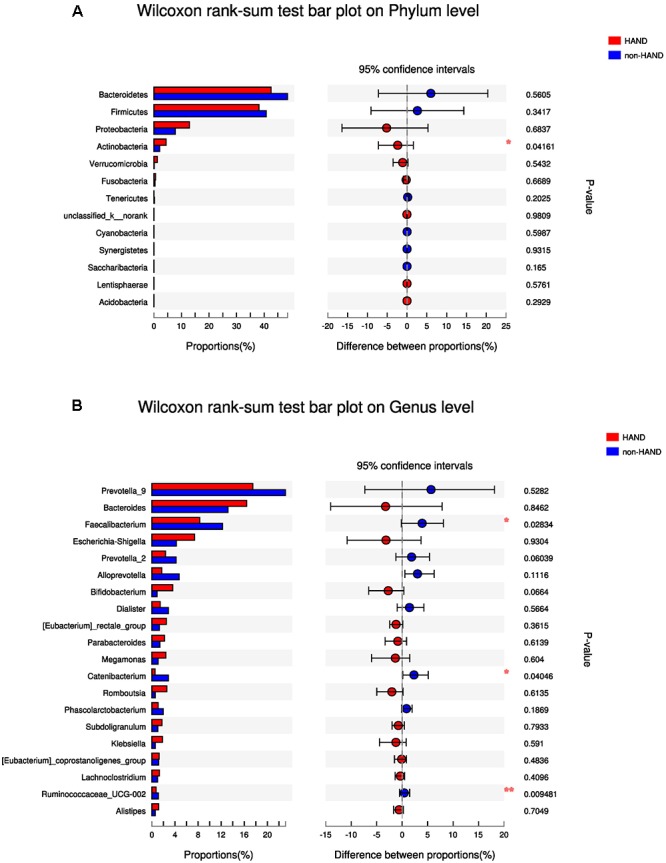
Difference in gut microbiota composition between the HAND group and the non-HAND group. **(A)** Wilcoxon rank-sum test bar plot on Phylum level. **(B)** Wilcoxon rank-sum test bar plot on Genus level. Each color represented a group, HAND group (red), non-HAND group (blue). ^∗^*P* < 0.05 and ^∗∗^*P* < 0.001. Confidence intervals were estimated using a percentile bootstrapping method.

At the phylum level, species abundance of *actinobacteria* was higher in the HAND group than in the non-HAND group (4.459 vs. 2.108%; *P* = 0.042; Figure 4A). However, this difference disappeared after FDR correction (adjusted *P* = 0.541). At the genus level, lower abundances of *Faecalibacterium* (8.304 vs. 12.23%; *P* = 0.028), *Catenibacterium* (0.552 vs. 2.877%; *P* = 0.040), and *Ruminococcaceae* (0.7261 vs. 1.138%; *P* = 0.009) were detected in the HAND group in contrast to the non-HAND group (Figure 4B). After for FDR correction, the differences in the abundance of *Faecalibacterium* (adjusted *P* = 0.625), *Catenibacterium* (adjusted *P* = 0.625), and *Ruminococcaceae* (adjusted *P* = 0.625) were not significant between the aforementioned two groups.

### Predictive Function Profile of the Gut Microbiota

**Figure 5:**
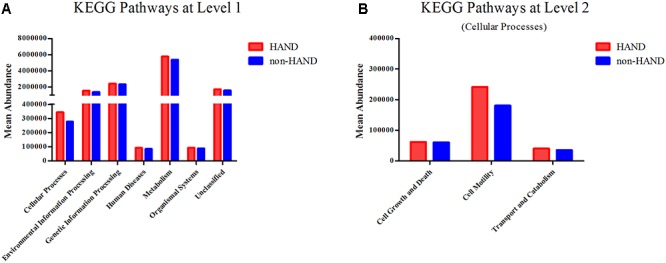
Functional predictions for the fecal microbiome of the HAND and non-HAND groups by KEGG (Kyoto Encyclopedia of Genes and Genomes) pathways. **(A)** KEGG pathways at level 1, *t*-test. **(B)** KEGG pathways at level 2, *t*-test.

Predictive functional profiling using the KEGG pathway showed that gut microbiota in the HAND group were associated with higher abundances in the cellular processes compared to the non-HAND groups (mean 344019 vs. 277003; *P* = 0.027). As microbiota function in cellular processes mainly include cell growth and death, motility, transport, and catabolism, we conducted a level 2 analysis of the KEGG pathways. After FDR correction, no statistical differences were detected between the two groups (Figure 5).

## Discussion

Gut microbiota dysbiosis has been linked to neurocognitive disorders in the HIV-negative population (Miklossy, 2011; Poole et al., 2013; Yang et al., 2016; Zhan et al., 2016; Noble et al., 2017). Nevertheless, an understanding of its role in the pathogenesis of HAND is still lacking to date. In the current study, we showed that gut microbiota dysbiosis is not independently associated with HAND. To the best of our knowledge, this is the first study to explore the association between gut microbiota and HAND.

We reported, along with others, that gut microbiota dysbiosis in PLWHA is common (Nowak et al., 2015; Carding et al., 2017; Desai and Landay, 2018; Lu et al., 2018; Missailidis et al., 2018). Gut microbiota from PLWHA showed significantly lower α-diversity compared with that from HIV-uninfected controls (Sun et al., 2016; Hamad et al., 2018). In this study, we found that microbiota in the HAND group also displayed significantly lower α-diversity than that in the non-HAND group, indicating associations between gut microbiota and HAND. Consistent with these results, a recent pilot study showed that supplementation with a probiotic containing *Streptococcus salivarius*, *S. thermophilus*, Bifidobacteria, *Lactobacillus* spp., and *S. faecium* improved neuropsychological performance in PLWHA (Ceccarelli et al., 2017). However, in our subgroup analysis adjusting for gender, age, education level, and CD4 T-cell count, all of which has been linked to HAND, the diversity and composition of the gut microbiota was comparable between the two groups in the current study (Wang et al., 2013). Moreover, no significant differences in the predictive function of the gut microbiota was found between the HAND and non-HAND groups. It is known that confounders such as age, gender, CD4 T-cell counts, and sexual preference also have significant effects on gut microbiota (Nowak et al., 2015; Ji et al., 2018). We and others have recently demonstrated that low CD4 T-cell counts, rather than HIV serostatus, predict the presence and recovery of gut dysbiosis in HIV-infected subjects (Guillen et al., 2018; Ji et al., 2018; Zhou et al., 2018). Furthermore, other studies found that MSM (men who have sex with men) had a significantly richer and more diverse fecal microbiota than non-MSM individuals, independent of HIV infection (Noguera-Julian et al., 2016; Kelley et al., 2017). Therefore, our results do not preclude the role of intestinal dysbiosis in the pathogenesis of HAND. However, our data do show that gut microbiota dysbiosis is not independently associated with HAND. Moreover, we do not know whether the compositions of the virome and fungal microbiome in the gut may contribute to HAND, which warrants further investigation.

Gut microbiota may contribute to neurological diseases by regulating both the production and absorption of neurotoxic products. Stimulation of the kynurenine pathway has been closely linked to the pathogenesis of HAND (Heyes et al., 2001; Smith et al., 2001; Valle et al., 2004; Vamos et al., 2009; Davies et al., 2010; Kandanearatchi and Brew, 2012; Vecsei et al., 2013; Myint and Kim, 2014). Indeed, even long-term ART in PLWHA did not normalize the overactivated kynurenine pathway (Chen et al., 2014, 2018). The levels of Lactobacilli and *Proteobacteria* in the gut have been correlated with kynurenine pathway activity and disease progression, respectively (Vujkovic-Cvijin et al., 2013; Zelante et al., 2013). Therefore, they might contribute to the development of HAND. However, no significant differences in these bacteria (Lactobacilli 0.044 vs. 0.046%, *P* = 0.710; *Proteobacteria* 12.94 vs. 7.79%, *P* = 0.684) were observed in our study.

Gut microbial dysbiosis has been associated with HIV pathogenesis (Vyboh et al., 2015; Dillon et al., 2016). Indeed, gut microbial dysbiosis is associated with many immunological parameters including T-cell activation and plasma lipopolysaccharide and soluble CD14 levels, all of which have been linked to disease progression (Dillon et al., 2014; Dinh et al., 2015; Nowak et al., 2015; Vazquez-Castellanos et al., 2015; Monaco et al., 2016; Noguera-Julian et al., 2016; Serrano-Villar et al., 2017; Vazquez-Castellanos et al., 2018). Moreover, interventions that modulate alterations in gut microbiota improved these markers in some clinical trials (Irvine et al., 2010; Gori et al., 2011; Kristoff et al., 2014). However, few studies have deciphered the association between microbiota and clinical diseases in HIV infection. Recently, altered microbiota dysbiosis has been linked to coronary heart disease, type 2 diabetes, and poor CD4 T-cell recovery (Hoel et al., 2018; Kehrmann et al., 2018; Lu et al., 2018). Unfortunately, these studies involved relatively small sample sizes and did not take into account other factors that significantly effects gut microbiota (e.g., CD4 T-cell count and sexual preference), which might bias their results. Interestingly, a landmark study recently showed no effect of experimental gut microbiota dysbiosis on disease progression in an SIV infection model, indicating that the associations we observed in PLWHA may result from the confounders (Ortiz et al., 2018). Therefore, we suggest that future microbiome studies on HIV infection should take these confounders into consideration (Noguera-Julian et al., 2016; Tincati et al., 2016; Ribeiro et al., 2017; Hamad et al., 2018; Klatt and Manuzak, 2018; Williams et al., 2018).

There are some limitations in our study that should be addressed. Firstly, the relatively small sample size might generate bias in our results. Secondly, in using MoCA as a reference for grouping, PLWHA exhibited a narrow score distribution interval, leading to a lack of HAD subjects. A recent study showed that the intestinal microbiota of people living with HAD was highly specific (Pérez-Santiago et al., 2017). Finally, all subjects we enrolled were ART naïve. Therefore, our results cannot be extended to PLWHA who have already been treated.

## Conclusion

In conclusion, our research shows that gut microbiota dysbiosis is not independently associated with neurocognitive impairment in PLWHA.

## Author Contributions

JC, JyS, and HL conceived and designed the study. FZ, JY, YJ, and JC acquired and analyzed the data. FZ, MS, and JjS assessed the cognitive status of the PLWHA. JW, LL, YS, and RZ were responsible for PLWHA recruitment. FZ performed the laboratory work. FZ and JY wrote the first draft of the manuscript. FZ, JC, and HL contributed to the final version of the manuscript. All authors read and approved the final manuscript.

## Conflict of Interest Statement

The authors declare that the research was conducted in the absence of any commercial or financial relationships that could be construed as a potential conflict of interest.
